# 452. Correlation of Charleston Comorbidity Index Score as the COVID-19 Pandemic Surged Throughout HCA Healthcare Facilities and Patient Outcomes

**DOI:** 10.1093/ofid/ofab466.651

**Published:** 2021-12-04

**Authors:** Irene Riestra Guiance, Ernesto Robalino Gonzaga, Isabel Riestra, Steven Char, Minh Q Ho

**Affiliations:** 1 UCF/HCA GME Consortium, Kssimmee, Florida; 2 UCF/HCA Healthcare GME, Kissimmee, Florida; 3 Suffolk University, Maitland, Florida; 4 Orlando VA Healthcare System, 14014 Deep Forest Court, Florida

## Abstract

**Background:**

As the COVID-19 pandemic raged throughout the United States, the healthcare system was strained due to a sudden increase in demand. Testing was initially limited, and the perception was that patients with high comorbidity burden were at higher risk for poor outcomes. The Charleston Comorbidity Index (CCI) is widely used as a predictor of prognosis and one-year mortality for a wide range of pathologies. This study aims to assess whether a correlation exists between CCI score, COVID-19 incidence throughout the pandemic and patient outcomes.

Charleston Comorbidity Index Score

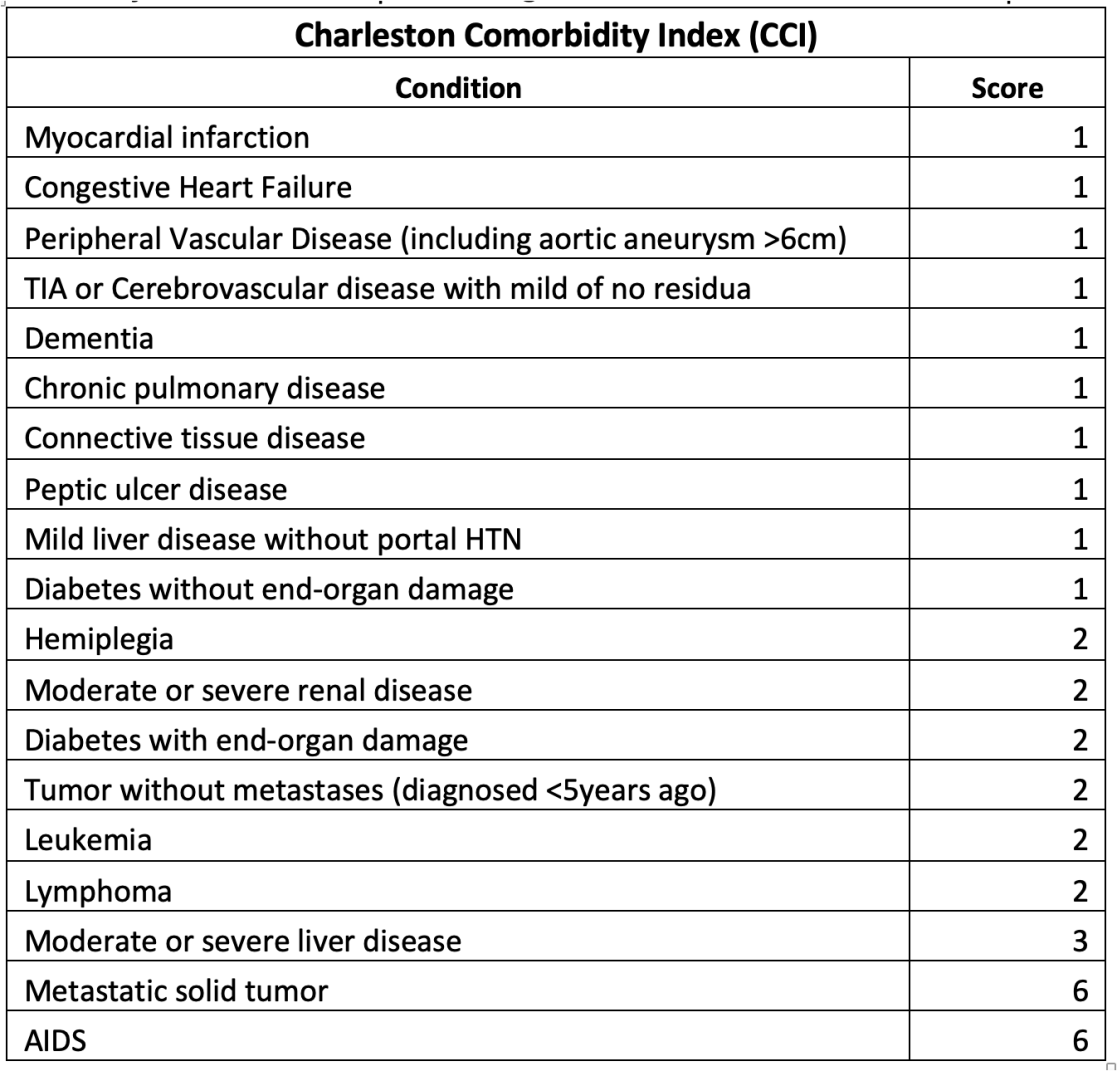

Scoring system for Charleston Comorbidity Index (CCI). Plus 1 point for every decade age 50 years and over, maximum 4 points. Higher scores indicate a more severe condition and consequently, a worse prognosis.

**Methods:**

Multicenter, retrospective review of patients diagnosed with COVID-19 from January 2020 to September 2020 throughout the HCA Healthcare system. The percent of total encounters that were COVID-19 positive by state was calculated along with the average CCI score for COVID-19 patients in 2-month increments. Patient outcomes were obtained across the entire population.

**Results:**

A clear surge of infected patients was seen in almost all states in the dataset from May 2020 onward except in Colorado and Louisiana where the percentage of COVID-19 positive encounters decreased until July 2020. As summer 2020 progressed, the highest percentage of COVID-19 positive encounters among HCA Healthcare facilities was in Florida and Texas. However, despite the fact that more patients were COVID-19 positive in these states, the CCI score was the lowest (Figure 1). The highest average CCI throughout the 9-month period was 7.66 in Colorado. In the first two months of the pandemic, patients who tested positive for COVID-19 had higher CCI scores on average than those who became COVID-19 positive later in the pandemic. Missouri had the lowest CCI average but the highest ICU admissions and in-hospital mortality. Indiana had the lowest average CCI score, and lowest admission rate (Figure 2).

COVID-19 Encounters and Average CCI score by State from January 2020 to September 2020

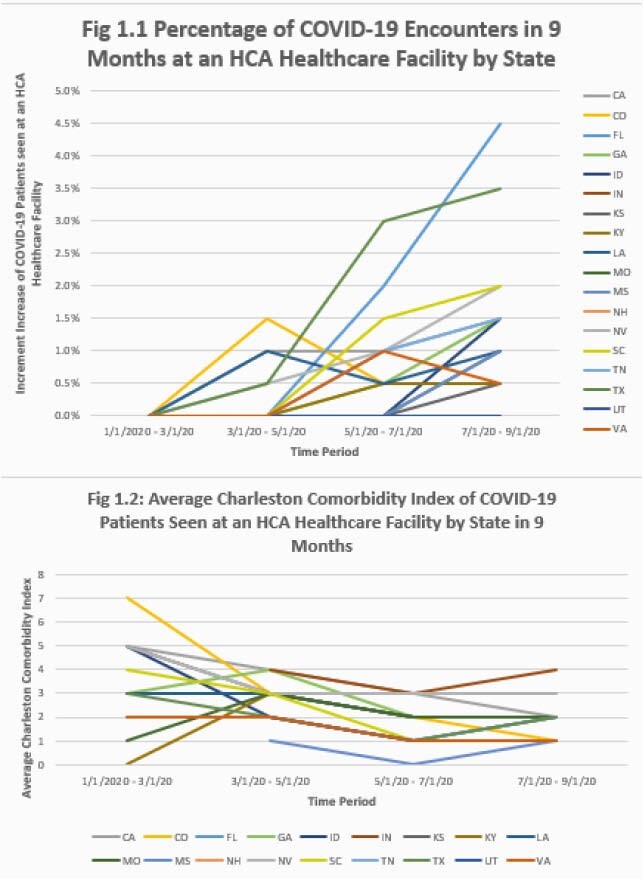

Graph 1: Percentage of COVID-19 Encounters in 9 Months at an HCA Healthcare Facility by State: Graph presents data obtained for the total of 92,800 patient encounters from January to September 2020 and recorded in 2-month increments. The rate of positive encounters throughout 18 states increased on average from May to September. From January to March 2020, the facilities with the highest rate of COVID-19 encounters were in Colorado, Louisiana and Texas. The states with the highest increment increase of COVID-19 positive patients were Texas, Florida and South Carolina and were trending up as the pandemic wore on through the summer of 2020. Graph 2: Average Charleston Comorbidity Index of COVID-19 Patient Seen at an HCA Healthcare Facility by State in 9 Months: In winter 2020 (January to March 2020) the average CCI score for patients seen with COVDI-19 was higher than in the Spring and Summer 2020 in all states except in Montana and Kentucky. Summer 2020 (May to July 2020) demonstrated some of the lowest average CCI scores for COVID-19 positive patients seen at an HCA Healthcare Facility.

Rate of Positive COVID-19, Patient Outcomes and Average Charleston Comorbidity Index Score by State

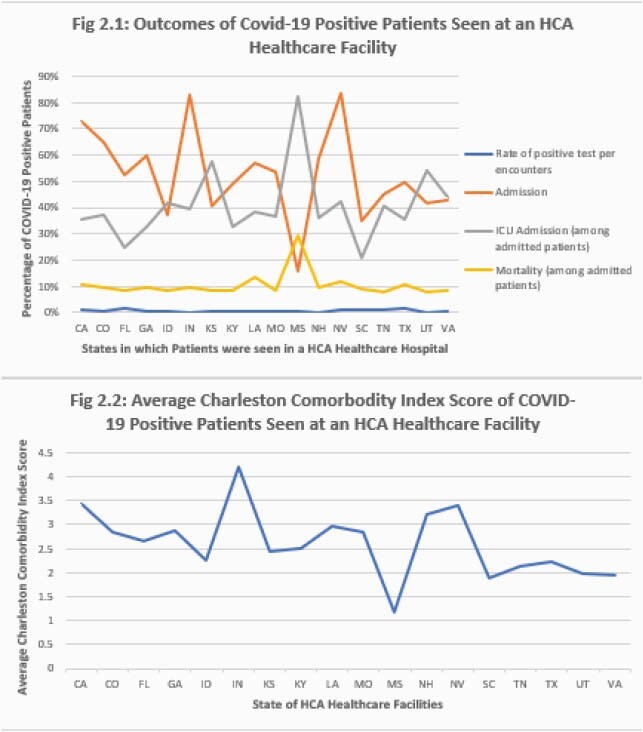

Graph 3: Outcomes of COVID-19 Positive Patients Seen at an HCA Healthcare Facility: Mortality and ICU admission was the highest in Missouri, however, the state had the least COVID-19 patients admitted. The rate of positive test per encounter was the highest in Florida and Texas. Texas had a higher mortality among admitted COVID-19 patients than Florida, however, Florida had a higher percentage of COVID-19 patients admitted. Graph 4: Average Charleston Comorbidity Index Score of COVID-19 Positive Patients Seen at an HCA Healthcare Facility: Average CCI was the lowest in Missouri. The states with the highest CCI score were Indiana, California, New Hampshire and Nevada.

**Conclusion:**

We observed an inverse correlation between CCI score and COVID-19 incidence while seeing that, on average, COVID-19 positive patients had higher CCI score in the first few months of the pandemic when incidence rate was lower. CCI score did not correlate to ICU admission, but a higher CCI score correlated to higher admission rate.

**Disclosures:**

**All Authors**: No reported disclosures

